# P-157. Efficacy and Safety of Helicobacter pylori Triple Therapy With and Without Saccharomyces boulardii Supplementation: An Updated Systematic Review and Meta-Analysis of Randomized Controlled Trials

**DOI:** 10.1093/ofid/ofaf695.381

**Published:** 2026-01-11

**Authors:** Omar Elkoumi, Ahmed Elkoumi, Mariam Khaled Elbairy, Hatim Nasruldin Shahin, Ahmad Beddor, Mostafa Adel T Mahmoud, Amr Khaled Elnegiry, Abdulqadir J Nashwan

**Affiliations:** Faculty of Medicine, Suez University, Suez, Egypt, Suez, As Suways, Egypt; Faculty of Oral and Dental Medicine, Egyptian Russian University, Badr City, Al Qahirah, Egypt; Faculty of Medicine, Suez University, Suez, As Suways, Egypt; Faculty of Medicine, South Valley University, Qina, Egypt, Qina, Qina, Egypt; Faculty of Medicine, Yarmouk University, Irbid, Jordan, Irbid, Irbid, Jordan; Faculty of Medicine, Beni Suef University, Beni Suef, Egypt, Bani Suwayf, Bani Suwayf, Egypt; Faculty of Medicine, Suez University, Suez, As Suways, Egypt; Department of Nursing, Hazm Mebaireek General Hospital HMGH, Hamad Medical Corporation HMC, Doha 3050, Qatar, Doha, Ad Dawhah, Qatar

## Abstract

**Background:**

The eradication of *Helicobacter pylori (H. pylori)* remains a significant challenge due to increasing antimicrobial resistance. *Saccharomyces boulardii (S. boulardii)*, a probiotic with anti-inflammatory and antimicrobial properties, has been proposed as a beneficial adjunct for *H. pylo*ri eradication. This meta-analysis evaluates the efficacy of *S. boulardii* supplementation to triple therapy in enhancing *H. pylori* eradication rates and reducing adverse events.

**Figure 1.**
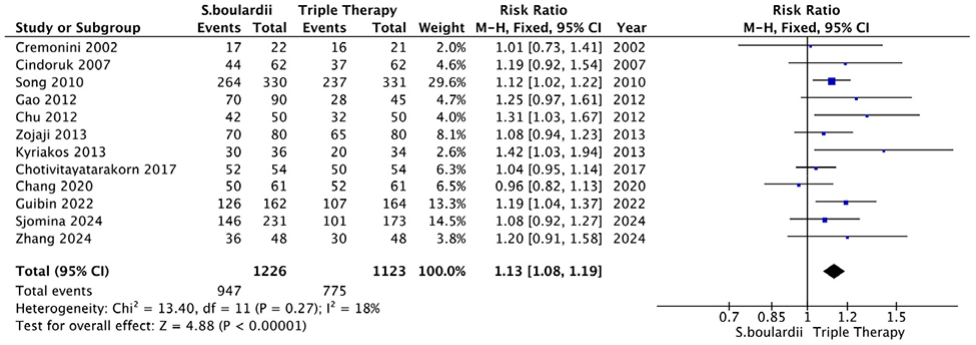
Forest plot of H. pylori eradication (ITT). CI, confidence interval; M–H, Mantel–Haenszel.

**Methods:**

We systematically searched PubMed, Embase, Cochrane Central, Web of Science (WOS), and Scopus databases from inception until March 2025. We included randomized controlled trials (RCTs) comparing individuals receiving *S. boulardii* supplementation alongside triple therapy versus triple therapy alone for *H. pylori* eradication. Statistical analyses were performed using Review Manager 5.4.

**Results:**

Thirteen RCTs encompassing 2,349 patients from diverse demographic backgrounds were included. Patients receiving *S. boulardii* as an adjunct demonstrated significantly higher *H. pylori* eradication rates compared to those on triple therapy alone (ITT analysis: RR 1.13, 95% CI [1.08, 1.19], p < 0.00001; PP analysis: RR 1.07, 95% CI [1.02, 1.11], p = 0.002). Additionally, *S. boulardii* supplementation significantly reduced the incidence of vomiting (RR 0.24, 95% CI [0.06, 0.98], p = 0.05), diarrhea (RR 0.40, 95% CI [0.32, 0.50], p < 0.00001), taste distortion (RR 0.72, 95% CI [0.55, 0.93], p = 0.01), abdominal distension (RR 0.67, 95% CI [0.48, 0.93], p = 0.02), and abdominal pain (RR: 0.53, 95% CI 0.36 – 0.79, P = 0.002).

**Conclusion:**

*S. boulardii* supplementation enhanced *H. pylori* eradication rates. Its addition to triple therapy reduced the incidence of diarrhea, vomiting, taste distortion, and abdominal distension compared to triple therapy alone. Further large-scale, multicenter trials are warranted to validate these results.

**Disclosures:**

All Authors: No reported disclosures

